# ‘They are lovely men’: Compassionate exclusion used to justify a protest outside asylum seeker accommodation

**DOI:** 10.1111/bjso.70045

**Published:** 2026-02-06

**Authors:** Alastair Nightingale, Sarah Jay

**Affiliations:** ^1^ REPPP, Centre for Crime, Justice & Victim Studies, School of Law University of Limerick Limerick Ireland; ^2^ Department of Psychology University of Limerick Limerick Ireland

**Keywords:** asylum seekers, critical discourse analysis, political protests, racism, violence

## Abstract

This study employed critical discursive and rhetorical psychology to analyse the discourses drawn upon to justify an arguably violent protest outside a previously disused hotel in rural Ireland, where 34 male asylum seekers had been accommodated. Interviews with protesters and public representatives were retrieved from three mainstream media platforms. The protesters drew on three contradictory and deracialized discursive strategies to inoculate their justification for the protest against accusations of prejudice, which we label compassionate exclusion. The first is a compassionate concern about the suitability of the accommodation for the asylum seekers, whilst engaging in collective action to force the asylum seekers into homelessness and risk of further violence. The second positions the protesters as compassionate towards the asylum seekers whilst demanding that they receive vetting and that the local community receive prior consultation on their suitability for accommodation. The third presents the ‘male’ asylum seekers as a threat to women in this isolated rural community, even though the protesters position themselves as compassionate towards the ‘lovely men’ who are already accommodated. This highlights how compassionate humanitarian concerns can be co‐opted to justify an arguably violent demand for the forced removal and exclusion of asylum seekers, whilst avoiding accusations of racism.

## INTRODUCTION

Violent opposition to asylum seeker accommodation has become an increasing concern across Europe. Hence, this article employed critical discursive psychology (Edley, 2001; Wetherell, 1998; Wetherell & Potter, 1992) and rhetorical psychology (Billig, 1987; Billig et al., 1988) to analyse previously unexplored discourses drawn upon to justify a protest outside an asylum seeker accommodation centre in rural Ireland. It drew on publicly available interviews in the media to explore the discourses used by protesters to legitimize their arguably violent demand for the forced removal and exclusion of asylum seekers. This study explored how these protesters presented an argument that avoids accusations of prejudice through a sanitized liberal discursive strategy of compassionate exclusion. Protesters claimed the accommodation was unsuitable for the asylum seekers, there was a lack of consultation from the government, and male asylum seekers were constructed as a particular threat to the women in this isolated rural community. Nevertheless, the protesters also consistently rejected the notion that they or their actions were in any way hostile towards the asylum seekers, who were described by one protester as ‘lovely men’.

This protest involved a round‐the‐clock roadblock outside a previously disused hotel, where 34 young, male, Black and Brown asylum seekers were accommodated on 15 May 2023. The protesters threatened the residents by driving tractors up to the accommodation centre in the middle of the night, forcibly preventing new arrivals by blocking roads leading to the accommodation, and boarding buses to count the number of residents leaving and returning (Boland, 2023; Dalton, 2023). Several intimidated residents left the accommodation on foot to return to Dublin, at risk of their asylum applications being closed, sleeping on the streets, and potentially facing further violence (Boland, 2023; Deegan, 2023). Eventually, the government abandoned the plan to increase the number accommodated at the hotel. Although the blockade ended on 21 May 2023, the protest continued outside the accommodation centre until the remaining 26 residents were moved on 12 July 2024 (Cooke, 2024).

This protest took place in Ireland, where there were rising hate crimes (Irish Human Rights & Equality Commission, 2024), increasing reports of racist assaults (Michael et al., 2022) and increasing violent attacks on asylum seekers (Power, 2023b). An acute shortage of accommodation meant rising numbers of male asylum seekers were resigned to sleeping on the streets of Dublin; they, along with the volunteers supporting them, were experiencing increasing violent assaults (Irish Refugee Council, 2024; Johns, 2023; Power, 2023b; Wilson et al., 2023). Protests outside asylum seeker accommodation centres were increasingly common (Cannon & Murphy, 2024), and arson attacks on buildings earmarked for asylum seeker accommodation, or rumoured to be, were rising (Lally & Fallon, 2023). This culminated in the worst anti‐immigrant violent rioting ever seen in Dublin in November 2023 (Power, 2023a). Although the protesters in the current study claimed their collective action was peaceful, forcing a group of people out of their home through intimidation and preventing others from accessing a home can only be understood as violent, especially when it puts the displaced people at further risk of violence.

### Discourses of violent exclusion

Arguably, collective violence is mobilized by constructing a category distinction between ‘us’ and a ‘dangerous, culturally alien’ other (Elcheroth & Reicher, 2017) and is often legitimized through expressions of self‐defence (Butler, 2020). In this regard, research has documented how arguments against refugee resettlement are constructed by linking them with fear and criminality (Figgou et al., 2011; Figgou & Condor, 2006) and a source of intergroup conflict (Zisakou & Figgou, 2019). And presenting immigrants as low‐skilled welfare scroungers (Gibson et al., 2018) and a threat to national culture (Gibson & Hamilton, 2011).

Similarly, politicians such as Kemi Badenoch in the United Kingdom (Solomon, 2025) and Donald Trump in the United States (Jacobs, 2018) perpetuate racist myths about immigrants being dangerous criminals and sexual predators (Chouhy & Madero‐Hernandez, 2020; Goodfellow, 2020; Ogungbure, 2018). Bennett (2025) convincingly shows that these anti‐immigrant myths, in the United Kingdom, are based on White supremacy, which is established through a history of colonial exploitation and violence.

However, research has also shown that discourses legitimizing discrimination tend not to be unitarily hostile but are saturated with ambivalent moral tensions and strategic denials of prejudice (Augoustinos & Every, 2007). Van Dijk (1992, 1993a, 1993b) identified the prevalent denial of racism, ‘I have nothing against Blacks but …’ (also see Condor et al., 2006; Hewitt & Stokes, 1975). Similarly, Wetherell and Potter's (1992) seminal work emphasized how speakers distance themselves from racism by constructing it as something done by problematic, pathological individuals, whilst positioning their own hostility towards minorities as reasonable and rational (also see Rapley, 2001; Verkuyten, 1998). Even though participants reject stereotyping refugees as criminals, they still present this stereotypical assumption that others hold as sufficient justification to exclude refugees (Figgou & Condor, 2006). Likewise, protesters explicitly opposing asylum seeker accommodation in Ireland denied being racist and right‐wing (Cannon & Murphy, 2024), and in the United Kingdom, sanitized their argument with the trope—‘we are not racist, just realist’ (Grillo, 2005).

Importantly, Billig (1991) argues that prejudice denial is not merely a means of impression management but a necessary rhetorical strategy to present a rational and warranted argument that avoids being disregarded as prejudiced and uninformed. An effective argument that legitimizes discrimination has to acknowledge and take up the ‘norm against prejudice’ by appearing balanced, thought‐through and avoiding explicit prejudice (Billig, 1991). He notes that prejudice denial can take place in many contexts, even when the speaker is in the presence of others who share their viewpoint, because it is an effective means to justify their position to themselves and others. Hence, the ‘norm against prejudice’ also affords the opposing ideological position—the social norm that sanctions the expression of prejudice (Billig, 1991)—and allows the speaker to articulate what could otherwise not be said (Augoustinos & Every, 2010).

Likewise, Augoustinos and Every (2007) highlight how speakers draw on ‘discursive deracialization’, which avoids using biological racial categories when constructing a minority negatively. Anti‐immigrant far‐right political parties have achieved increasing electoral success by shifting their ideological position from ‘biological racism’ to sanitized ‘cultural racism’ (Rydgren, 2005). Twitter posts present Ireland as progressive and inclusive (Sambaraju, 2022), and White Irish interviewees denied that the Irish are racist (Sambaraju & Minescu, 2019). Arguably, this ‘not racism’ discourse is in itself ‘racist violence’ because the powerful White majority withhold the definition of racism from those who experience it (Lentin, 2018).

Furthermore, accusing a speaker of being racist is often seen as a ‘more serious social infraction than racist attitudes or actions themselves’ (van Dijk, 1992). Hence, speakers who attempt to challenge racist talk are likely to seriously jeopardize their relationship with others who are present (Condor, 2006). This has profound implications for attempts to call racist talk to account (Augoustinos & Every, 2010). Those who take up anti‐immigration discourse often claim that freedom of speech is being suppressed by accusations of racism (Goodman, 2010; Lentin, 2018). Powerful majority speakers accused of prejudice often claim they are being discriminated against (van Dijk, 1992; Verkuyten, 1998) and marginalized from mainstream politics (Rooyackers & Verkuyten, 2012). Every and Augoustinos (2007) highlight that Australian parliamentarians who defend asylum seekers, cautiously navigate their arguments and can be disciplined for accusing opposition speakers of racism. Speakers who are sympathetic towards asylum seekers struggle to present politically contentious arguments demanding unconditional inclusion and instead resort to ambivalent paternalism, which focuses on their shared emotional distress (Nightingale et al., 2017). Lynn and Lea (2003) highlight how advocates on behalf of asylum seekers were constructed as affluent White liberals who do not shoulder the economic burden.

Liberalism displays an ‘ideological dilemma’ (Billig et al., 1988), a moral tention between, on the one hand, tolerance and the inclusion and, on the other hand, the universally accepted notion that nation‐states have the moral right to exclude unwanted others and are the legitimate arbitrators of violence (Billig, 1995; Butler, 2020). Hence, state‐sanctioned violent exclusion of those seeking asylum is legitimized through prejudiced denials and liberal ideologies (Andreouli & Dashtipour, 2014; Augoustinos & Every, 2007; Durrheim et al., 2015; Goodman, 2008; Goodman & Burke, 2010). When it comes to the contentious issue of immigration, the norm against prejudice is malleable and utilized to legitimize exclusion (Every, 2013; Goodman, 2010). These prevalent denials of prejudice and limited definition of racism to biological criteria allow the supposed non‐racist majority to present their hostility towards asylum seekers as pragmatic and commonsense.

Violent exclusion is cloaked in rational pragmatism, and liberal discourses emphasizing fairness are mobilized to present asylum seekers as an unreasonable burden on employment, housing and social services (Goodman & Kirkwood, 2019; Wodak, 2012). Condor et al. (2006) showed how prejudice denial can be a ‘collaborative accomplishment’ where speakers defend others from accusations of racism and construct them as decent and reasonable citizens. Far‐right nationalist politicians take up transnational discourses claiming asylum seekers receive preferential treatment and undermine the true cultural diversity between monocultural nation‐states (Nightingale et al., 2021), and they blame immigrants for increasing inequality (Jay et al., 2019). They present themselves as rational actors who singularly have the political ability to defend the interests of the majority, homogeneous ‘people’ against unwanted culturally diverse immigrants (Mols & Jetten, 2014; Rooyackers & Verkuyten, 2012; Sakki et al., 2017; Sakki & Pettersson, 2016; Wodak et al., 2013). Right‐wing speakers in the Australian parliament positioned themselves as ‘ordinary Australians’ (Rapley, 1998, 2001) and Geert Wilders in the Netherlands positioned himself as a ‘responsible and realistic politician’ (Rooyackers & Verkuyten, 2012). Politicians work up rational discourses claiming it is common sense to restrict immigration (Capdevila & Callaghan, 2008).

Speakers present the exclusion of asylum seekers as rational and warranted by taking up an entitled category position as citizens who have the moral right to exclude unwanted others and defend the sovereignty of the nation (O'Doherty & Augoustinos, 2008). National belonging is deployed to categorize, us and them, which promotes hostility without explicit appeals to race (Wodak, 2012). National nostalgia for a glorious past is also the basis of emotive anti‐immigrant arguments (Mols & Jetten, 2014). Even though, as Goodfellow (2020) argues, hostility towards asylum seekers and immigrants is based on entrenched assumptions of White supremacy—White, Christian and civilized versus Black, Muslim and barbaric.

Lynn and Lea's (2003) pivotal article shows that the categorization of some asylum seekers as bogus has become embedded in hegemonic discourse and casts suspicion on all asylum seekers. This taken‐for‐granted notion that the host nation's humanitarianism and tolerance are being exploited was evident in the talk of UK citizenship officers (Andreouli & Dashtipour, 2014) and in Australian debate (Every, 2008; Every & Augoustinos, 2008). Categorizing asylum seekers as ‘illegal immigrants’, ‘economic migrants’ and ‘boat people’ was used to justify harsh treatment (Goodman & Speer, 2007; O'Doherty & Lecouteur, 2007). Hostility towards the integration of asylum seekers can be mobilized by categorizing them as ‘aggressive Muslims’ and a threat to the host nation's culture (Goodman & Kirkwood, 2019).

Although there has been extensive work exploring anti‐immigrant political discourse, some of which has been reviewed here, there has been limited investigation of discourses drawn upon to justify violent protests outside asylum seeker accommodation (for exceptions, see Cannon & Murphy, 2024; Grillo, 2005). This study makes an important contribution by exploring how speakers managed their arguments, justified their collective action of intimidation, forced removal and exclusion of asylum seekers. It explores how protesters potentially avoided accusations of prejudice and positioned the justification as rational and warranted. This extends the understanding of contemporary racism and denial, and of justifications for the violent, enforced removal and exclusion of unwanted others. This is an urgent endeavour given the increasing hostility and violence towards racialized asylum seekers in wealthy Western liberal democracies (Goodfellow, 2020).

## METHOD

### Context

Ireland's Direct Provision system, established in 2000, is a profiteering form of open detention (Grayson, 2020), often using disused hotels where asylum seekers, including families with children, languish for many years. This system has been condemned by the United Nations (United Nations, 2015) and criticized for violating basic human rights due to substandard, overcrowded accommodation and humiliating conditions (Breen, 2008). Nevertheless, on 15 May 2023, 34 male asylum seekers were transported from Dublin to Magowna House in the west of Ireland with the plan to accommodate a further 69 people. For a week following their arrival, protesters blocked roads with tractors, silage bales and bollards.

### Data

All publicly available online audio and video recordings discussing the protest outside Magowna House were retrieved from three mainstream media outlets: Raidió Teilifís Éireann (RTE; national public service broadcaster), Clare FM (independent local radio) and The Irish Times (print media with online news videos and podcasts) (see Table S1 for more details). The total duration of the data was 4 h, 39 min and 58 s. These three media outlets were selected because they were the main source of information regarding the protest, with reporters providing interviews with the protesters and their representatives. Specifically, the local radio station, Clare FM, served as a conduit for protesters and their representatives to justify their actions. Audio and video recordings were selected because they allowed the analysis to interrogate the social interactions and also confirmed that speakers were not misquoted. This data is publicly available, and therefore, this study did not require approval from an institutional ethics committee.

The protesters were a fluid collective from the sparsely populated rural community surrounding Magowna House Hotel, who labelled themselves ‘The Concerned Residents of Inch’. Census data from 2022 indicates a total population of 424 people within approximately a 2 km radius, of which 95% identify as White Irish and 3% as White other; one person identified as Asian. Eight‐five percent identify as Catholic, and 78% indicate an occupation that can be considered middle class or above (Central Statistics Office, 2022). We make no assumptions about the protesters' political leanings or their affiliation with political organizations.

### Procedure

The first author transcribed the data. Then, both researchers repeatedly listened to the audio and read and reread the transcripts until both were fully immersed in the data. Initial thoughts, observations and points of interest were discussed and compiled into a document. One researcher coded the data to further interrogate what was being accomplished in the talk and explored patterns and exceptions. Particular attention was paid to justifications for the protest, and based on this, extracts of interest were selected and moved to a separate document for further discussion. Three discursive strategies were determined, along with the strongest supporting example extracts, which received fine‐grained analysis and are discussed in this article. This was a collaborative and iterative process.

The analysis was informed by critical discursive psychology (Edley, 2001; Potter & Wetherell, 1987; Wetherell, 1998; Wetherell & Potter, 1992) and rhetorical psychology (Billig, 1987, 1991). Hence, we approached the data by exploring discursive patterns (Wetherell, 1998, 2007)—recurrent, culturally shared ways of making sense of the world—and identity management and constructions of social categories. We focused on the action orientation of the discourse, what was accomplished through the accounts—reports or descriptions of an event or experiences—drawn upon by these speakers. Specifically, in order to answer the research question, we homed in on how ideological and subjective positions were managed and what discursive strategies—arguments used to justify the actions of the protesters—were taken up. We also paid attention to potential alternative explanations and counterarguments that were left unsaid (Billig, 1999) and to absences (Bennett, 2025). Importantly, the critical analysis aimed to reveal entrenched power relationships and inequities that were legitimized and contested through discourse (Wetherell, 1998), and in this regard, the discourse was interpreted within the broader social, cultural and historical context.

The analysis also drew on Wetherell's (2012, 2013) concept of affective–discursive practice, which interrogates the performance of recognizable affective patterns and routines that are culturally prescribed and evident in social interaction. This aided our interrogation of how affect and discourse were entangled and assisted our interpretation of affective patterns in political discourses. It questions what is being accomplished through the use of affect and explores how affect is strategically deployed in discourse to mobilize ‘affective alliances’ (Nightingale et al., 2017) and legitimize ideological positions. Specifically, how talk shapes and is shaped by affect, particularly within power relations and justifications of exclusion.

### Reflexivity

We are privileged, White and culturally similar immigrants. Hence, we have some limited experience of being outsiders, but have never suffered the hostility directed at asylum seekers and racialized minorities or the traumatizing journeys and insecurity that asylum seekers face. However, we unreservedly sympathize with their plight and take an emancipatory, social justice orientation and a social constructionist perspective. We hope this article will positively impact future discourse, policy and the material situation for asylum seekers.

## ANALYSIS

This analysis highlights three intertwined discursive strategies used to justify the action of the protesters, which we refer to as ‘compassionate exclusion’. The primary accomplishment of this discourse was to circumvent accusations that the protesters' collective action was motivated by prejudice, racism and hostility towards the asylum seekers. The first strategy constructs a compassionate concern for the asylum seekers being accommodated in *Unsuitable Accommodation*. The second maintains a compassionate position by diverting the justification for the protest away from an accusation of hostility towards the asylum seekers to a *Lack of Consultation* from the government. The third, *Men Causing Fear*, deracializes the demand to remove and exclude these asylum seekers by focusing on their gender and the risk these potential sexual predators pose to the local community. However, even in this strategy, the speakers maintain a position of compassion towards the asylum seekers who are already resident, and one speaker describes them as ‘lovely men’.

### Unsuitable accommodation

Central to the justification for the protest was the unsuitability of the accommodation for the asylum seekers. This compassionate concern was presented regularly by the protesters and their public representatives as a reason for blocking the roads.

#### EXTRACT 1: RTE RADIO—MORNING IRELAND 16/05/2023 PROTESTER SPEAKING

1. *I don't think there's any ill will to the to the people that have*
2. *arrived here today (↑) uhm certainly not I think we're probably*
3. *frustrated with the government at this stage (.) it is eight kilometres*
4. *to the centre of Ennis from here you know you have to travel nearly*
5. *six kilometres before you find the footpath (↑) so you know again (.)*
6. *what are these people going to do (↓) how are they going to*
7. *spend their time (↓) how are they going to be taken care of (.)*

Extract 1 shows how the protester carefully managed accountability and deflected potential accusations of prejudice (Augoustinos & Every, 2007; Billig, 1991; van Dijk, 1992) by constructing the protesters as not holding any hostility towards the asylum seekers, ‘I don't think there's any ill will to the people that have arrived here today’ (Line 1). This moral marker—‘no ill will’—explicitly distanced the protesters from accusations of racism, which was underscored with ‘certainly not’ (Line 2). The protester worked up an affective‐discursive position (Wetherell, 2013) of ‘frustration’ to justify their action and direct the cause of this frustration at ‘the government’ (Line 3).

The protester proceeded to outline that this frustration with the government was due to the isolated rural location of the accommodation, its distance from Ennis, the nearest town, and the lack of footpaths in the area (Line 4). These factual statements functioned as empirical grounds that anchored the speaker's concerns in logistical realism rather than emotion (Fairclough, 2003; Potter, 1996). However, the speaker also constructed a compassionate concern for the asylum seekers, using a three‐part list (Jefferson, 1990)—‘what are these people going to do (↓) how are they going to spend their time (↓) how are they going to be taken care of (.)’ (Line 6). Hence, the problem was framed as an infrastructural issue and certainly not a racial one. The location was unsuitable for the asylum seekers to have a reasonable, basic quality of life, which implied an affective position of compassionate exclusion.

#### EXTRACT 2: CLARE FM 19/05/2023 PROTESTER INTERVIEWED BY A REPORTER

1. *Reporter: But can you understand the view of some who think that*
2.     
*the approach you have taken is not the right one, that regardless of*
3.     
*Magowan House Hotel in the area is being unsuitable that these men*
4.     
*have escaped terrible situations at home, that they have to be housed*
5.     
*somewhere and at times it may be a less than ideal locations or*
6.     
*facilities (.)*
7. *Protester: Well, I mean at the end of the day everyone is entitled to*
8.     
*have a house (.) right (↑) that's the thing (.) and that's the bottom line*
9.     
*here (.) ahh the issue with the whole situation is that these people are*
10.     
*being placed, are placed in an unsafe, unsafe situation for*
11.     
*themselves, for everybody involved and that's the bottom line we do*
12.     
*not have an issue with people being supplied with houses that would*
13.     
*not be true*.

Extract 2 is another protester speaking on the local radio station, Clare FM, 3 days after Extract 1 and after representatives had met with the Minister of State. Notably, the protesters' position on the suitability of the accommodation was unchanged even after reassurances that support services for the asylum seekers would be put in place. The reporter questioned the legitimacy of this position (Line 1), and in response, the protester acknowledged that ‘everyone is entitled to have a house’ (Line 7) and underpinned this claim with a tag question ‘right’ (Line 8). The speaker was not expecting an answer to the question—‘right’—but was making the listener aware that they shared the same stance on this issue (Edwards & Potter, 1992). The speaker's support for the right to a home is unquestionable, and the justification for blocking access to the accommodation and demanding the removal of the asylum seekers is based on a concern for them being in an ‘unsafe situation’ (Line 10). The protester reiterated their compassionate position and corrected the reporter's accusation, ‘we do not have an issue with people being supplied with houses that would not be true’ (Line 12).

Previous discursive analysis has extensively highlighted how speakers deracialize their arguments for excluding asylum seekers by drawing on structural and economic reasons such as pressure on social services, employment and housing (see Augoustinos & Every, 2007; Capdevila & Callaghan, 2008; Condor et al., 2006; Every & Augoustinos, 2007; Goodman & Burke, 2010; Rapley, 2011; van Dijk, 1992, 1993b). Here, the speakers justify exclusion on compassionate grounds—a concern about the unsuitability and safety of the accommodation, primarily due to its isolated rural location.

However, it is noteworthy that the protester also specified that the ‘unsafe situation’ (Line 10) was not only for the asylum seekers but was ‘for everybody involved’ (Line 11). This implicitly orients to the third discursive strategy—men causing fear—that works up the specific category of male asylum seekers as a threat to the community. But first, we discuss the discursive strategy justifying the protest as a lack of consultation from the government.

### Lack of consultation

These protesters commonly justified their actions by referring to the lack of consultation from the government. This strategy is presented as an institutional failing and injustice experienced by the ‘concerned residents’, who positioned themselves as having category entitlement (Potter, 1996) to vet and decide on who can reside in their community.

#### EXTRACT 3: CLARE FM 19/05/2023 PROTESTER INTERVIEWED BY A REPORTER

1. *Reporter: But can you understand Anne‐Marie how when the international*
2.     
*protection applicants themselves arrived how the whole scenario*
3.     
*could have unsettled them and made them think it was about them (.)*
4. *Protester: Uhm (.hh) I I we can all understand the particular situation right (.)*
5.     
*the point is that (.) just a sec (.) there was absolutely zero consultation*
6.     
*and I mean (.) really and truly (.) that I thought that was a huge*
7.     
*factor in this whole situation I mean to think that (.) you know we*
8.     
*ask I I I'm kind of stuck for words in the whole situation because*
9.     
*basically we there was nobody consulted there was nothing arrived*
10.     
*(.) with announcement basically listed on Clare FM I mean it was it*
11.     
*it I suppose (↑) how would you say it (.) it would not have happened*
12.     
*if they're having a consultation because you know there would have*
13.     
*been a different approach people would have (.) had some chance to*
14.     
*see what was going on have an idea (.) yeah it was a reaction (↑) and*
15.     
*that's the way to put it (.) a reaction to something that was (.) it's*
16.     
*incredulous to be honest with the situation*

Extract 3 is of interest because the protester takes up a range of discursive devices to avoid the accusation that the asylum seekers may have felt intimidated by the protest. Whilst the reporter invoked an empathic framing that avoided explicitly accusing the protesters of prejudice. He cautiously hedged his question using ‘could’ (Line 3) instead of ‘would’ and minimized the potential distress caused—‘unsettled them’ (Line 3).

Nevertheless, this claim disrupted the protester's footing (Potter, 1996) as her speech was rife with hesitations and false starts. There was an audible sharp intake of breath as she stumbled to find the right words (Line 3), eventually delivering the rather ambiguous statement—‘we can all understand the particular situation right’ (Line 4). Through the use of the first person plural ‘we’, the speaker stated an indisputable shared understanding that the reporter was invited to agree on—‘right’ (Edwards & Potter, 1992). But the ‘particular situation’ was ambiguous, and the protester attempted some repair work to clarify the position—‘the point is that’ (Line 5). The protester stumbled again, and there was a brief break—‘just a sec’.

Finally, the protester regains her footing and uses an extreme case formulation to redirect the conversation away from the accusation—‘there was absolutely zero consultation’ (Line 5). This shifts attention away from a hostility towards the asylum seekers to a procedural injustice, a failure in the government's decision‐making process. The speaker drew on further rhetorical emphasis to construct sincerity and stake investment (Potter, 1996)—‘I mean really and truly’ (Line 6) and ‘I'm kind of stuck for words’ (Line 8). Additional extreme case formulations were deployed to further emphasize the injustice (Lines 6 and 9). And fact was constructed by using a concrete example that the government showed no more concern for local community consultation than any radio listener (Line 10).

The protester proceeded cautiously by hedging (Edwards & Potter, 1992)—‘how would you say’ (Line 11). She monitored her talk due to a sensitive and potentially controversial topic being discussed. A secure footing was eventually taken up, declaring that if they had received some prior consultation from the government, the protest ‘would not have happened’ (line 11) and ‘there would have been a different approach’ (Line 12). The protester adds, ‘they would have had some chance to see what was going on’ (Line 13), and they would ‘have an idea’ (Line 14). In an attempt to resolve the justification for the protest, which avoids the accusation of prejudice, she resorted to ‘yeah it was a reaction’ (Line 14). Exactly how this foresight and prior knowledge would have changed the local community's response was left unsaid (Billig, 1999). But the justification for the protest was inoculated against accusations of prejudice through an ambiguous downgrading to a mere response to a communication failure.

The protester displayed sudden insight and explicitly stated that she had now found the right words—‘that's the way to put it’ (Line 15). This worked to add credibility to her position as she appeared to be actively evaluating her stance within the interaction. This implied that the protest was spontaneous and not a pre‐planned or malicious act, but rather an inevitable response to an unjust situation, which mitigated accusations of intent to harm. The extract closed with an affective stance of shock and disbelief—‘incredulous to be honest’—constructing the protesters as rational but put in an untenable position. This emotional response was rooted in a perceived failure of governance and not a hostility towards asylum seekers. The protesters were presented as victims, not aggressors, and the potential distress caused to the asylum seekers is sidestepped and disregarded.

This speaker struggled to explain what she repeatedly referred to as the ‘situation’, but managed to avoid the reporter's accusation of hostility and to maintain the protesters' compassionate position. She made many false starts, hedging, stake inoculation and, importantly, much is left unsaid (Billig, 1999). Although the talk was cautious and hesitant, it effectively avoided capitulating to the accusation that the protest could be intimidating for the asylum seekers, even though the protesters were explicitly demanding their removal. However, this speaker does not explain why the lack of consultation was problematic for the protesters or how it would have changed their approach. The next discursive strategy offers insight into what was left unsaid by highlighting that the lack of consultation did not allow the local community to assess the suitability of the male asylum seekers to reside in the area.

### Men causing fear

Although the protesters expended significant discursive labour on working up a claim that they were not hostile to the asylum seekers, at the same time, they justified the enforced removal and exclusion by presenting the asylum seekers as a significant safety concern, particularly for women. In contrast to previous research showing how speakers employ fear of criminality (e.g., Figgou et al., 2011; Figgou & Condor, 2006) and inevitable intergroup conflict (Zisakou & Figgou, 2019) as justifications for excluding asylum seekers, these protesters made gender salient. Although they did not explicitly present the asylum seekers as potential perpetrators of sexual violence, the threat to the community was presented as a commonsense understanding that required little explanation other than a threat from a group of unvetted men with little to do. This concern was presented as particularly problematic for women in an isolated rural area which lacks streetlights, footpaths and phone coverage. The listener was left to determine exactly what form this threat takes. Even though the protesters presented the asylum seekers as a significant threat to the women in the community, they still maintained a compassionate position and claimed they did not hold any hostility towards the asylum seekers.

#### EXTRACT 4: RTE DRIVETIME 18/05/2023 PROTESTER INTERVIEWED BY A REPORTER

1. *Protester: they are lovely men*
2. *Reporter: would you be happy for them to stay I think that's the issue and I*
3.     
*know people want services and I know they're not here yet but these people*
4.     
*do need somewhere to live and there are no places it would seem (.)*
5. *Protester: if it was fit for purpose we would have got a consultation (.) and we*
6.     
*would have been involved in it and me and my family who are the local*
7.     
*people, we're very welcoming people and our community would have done*
8.     
*everything to integrate everybody in and we still will do everything to*
9.     
*integrate everybody in, we will do that but at the same time we do have*
10.     
*concerns as well that weren't addressed not nothing to do with the lads in*
11.     
*Magowna nothing to do with them but it by our government (.) so first of all*
12.     
*we need to know we need to know a vetting situation we need to know that we*
13.     
*have*
14. *Reporter: people are not vetted when they move to your community I was*
15.     
*not vetted to come here, anyone who moves in next door to you will not be*
16.     
*vetted that doesn't happen (.)*
17. *Protester: but it should happen(↑)*
18. *Reporter: you believed that it should happen okay (.) and the level of*
19.     
*consultation you're talking about I know it will be good but I've been in many*
20.     
*towns where these protests have happened and they don't those communities*
21.     
*are not consulted um*
22. *Protester: but is that okay (.) this is the point that we're putting across and the*
23.     
*other side of it is that we our community here I know you've probably noticed*
24.     
*we have absolutely no phone coverage (.) nothing we don't have streetlamps*
25.     
*because we don't have streets (speaker sounds upset)*
26. *Reporter: you're right yeah*
27. *Protester: we have absolutely nothing here (.) so every one of us here are*
28.     
*around looking for signal there's nothing happening and at the end of the day*
29.     
*there is going to be sixty‐nine males in here whether they're Irish whether*
30.     
*they're whatever nationality sixty‐nine men is a serious consideration for our*
31.     
*local community and it is a fear*

Extract 4 was an interaction between a reporter and a protester at the blockade, where the protester worked up an affectionate description of the asylum seekers as ‘lovely men’ (Line 1). This operated as a stake inoculation (Potter, 1996) as the protester was not motivated by hostility towards the ‘lovely’ male asylum seekers. But as the interaction unfolded, the protester's compassionate position became increasingly ambivalent, and she was reticent about offering sanctuary to male asylum seekers because they were a threat to the women in the community. The reporter sought clarity on the protester's position by asking, ‘would you be happy for them to stay’ (Line 2) and emphasized how this was a central concern for the asylum seekers—‘I think that's the issue’ and ‘these people do need somewhere to live’ (Line 4). It is worth noting that the reporter avoided the gender category and instead used the inclusive superordinate category ‘people’. Also, the reporter pre‐empted the unsuitable accommodation argument by highlighting that desirable support services were not yet in place, but this did not detract from the need for accommodation (Line 3).

However, the protester circumvented this argument by stating that the accommodation was not fit even for this basic purpose (Line 5). This was constructed as a fact (Potter & Wetherell, 1987) because if it was fit for purpose, the local community would have been consulted prior to the asylum seekers' arrival (Line 5). The protester proceeded to construct their compassionate position by claiming they were willing to ‘integrate everybody’ (Line 8). But this was immediately followed by an exception to the extreme case inclusion of everybody—‘but at the same time we do have concerns as well that weren't addressed’ (Line 9). This positioned the local community as having ‘category entitlement’ to demand prior consultation (Potter, 1996) whilst maintaining a compassionate stance.

This was followed by a further stake inoculation, doing additional preparation to mitigate against accusations of prejudice, ‘nothing to do with the lads in Magowna, nothing to do with them but it by our government’ (Line 10). The ground was carefully laid for the speaker to deliver the primary justification for the protest that the government should ‘vet’ male asylum seekers and consult with the local community on their suitability for accommodation (Line 12). Notably, this controversial position was delivered cautiously with false starts—‘so first of all we need to know we need to know’ (Line 12).

The reporter refuted this claim by stating that it is not normal procedure to vet people before they enter or take up residence within a community (Line 14). After a brief silence, the protester gave a definite response—‘but it should happen’ (Line 17). Although the asylum seekers were initially presented as ‘lovely men’, and much discursive work was done to present the local community as compassionate, the speaker also displayed explicit hostility towards their suitability to live in the locality. Interestingly, this speaker worked up a category entitlement—the local community—and positioned it on the high moral ground as compassionate and welcoming, but also having the moral right to demand the removal and exclusion of unwanted others. This is similar to the universal taken‐for‐granted assumption that the nation has the moral right to exclude unwanted others (Billig, 1995), but at a local community level.

The reporter proceeded to probe the community's right to demand prior consultation by making the comparison that other communities do not receive this (Line 18). The protester now appeared sure of her account, ‘but is that okay’, and emphasized that this issue of prior consultation was a central concern for the protesters—‘this is the point that we're putting across’ (Line 22). The protester proceeded to reify why this absence of prior consultation invoked a concern about the safety of women because of the poor infrastructure—‘you've probably noticed we have absolutely no phone coverage (.) nothing we don't have streetlamps because we don't have streets’ (Line 23). This implicitly worked up women's vulnerability and presented asylum seekers as potential perpetrators of sexual violence.

At this point, the protester expressed an affective position (Wetherell, 2012) of distress within the interaction as she audibly struggled to hold back her tears, which appeared to disarm the reporter and left him unable to deny or question the significance of the lack of phone coverage—‘you're right yeah’ (Line 26). The protester then, without prompting, proceeded to explain why this was an issue by repeatedly highlighting gender and the lack of infrastructure. This was followed by an inoculation against accusations of racism by claiming it does not matter what nation these ‘men’ come from; they could be Irish or ‘whatever nationality’ (Line 30). In sum, the protester worked up an ambivalent affect‐discursive position (Wetherell, 2003, 2012) torn between fear and compassion—‘Sixty‐nine men is a serious consideration for our local community, and it is a fear’ (Line 30), even though the men were previously described as ‘lovely’ (Line 1).

#### EXTRACT 5: RTE MORNING IRELAND 16/05/2023 PROTESTER SPEAKING

1. *I think if it was if if if you said to me there was uhh thirty families*
2. *coming here with kids uhm mums and dads I don't think you know*
3. *there would be any barriers (.) at all you would see the residents of*
4. *Magowna going up there and you know with toys maybe clothes if*
5. *needed you know welcoming them and we most certainly would um I*
6. *don't want anyone to think that we're here because you know um*
7. *we're we're hostile in any way (.) as as a lady I would feel*
8. *intimidated by ten men (.) standing (.) on the roadside or standing*
9. *where I walk at home (↑) in my own town (↑)*

In Extract 5, another protester described her affective response to men being accommodated in the area and contrasted this with how the local community would welcome the arrival of families (Line 3). This stake inoculation positions the protesters as rational and compassionate because there would be no ‘barriers’ (Line 3) to their welcoming of families. This was followed by an undeniable assertion of the collective moral stance (Potter, 1996)—‘we most certainly would’ (Line 5)—which pre‐empted any criticism about the community's ability to welcome outsiders. Even though the protesters were explicitly demanding the removal and exclusion of male asylum seekers, they managed to maintain a compassionate position and inoculate against accusations of prejudiced—‘I don't want anyone to think that we're here because you know um we're we're hostile in any way’ (Line 6). The speaker has prepared the ground for the controversial and sensitive statement to come.

The speaker constructs a hypothetical affective scenario making women's risk from sexual violence salient, ‘as a lady I would feel intimidated by ten men (.) standing (.) on the roadside or standing where I walk at home (↑) in my own town (↑)’ (Line 7). This affective position—‘as a lady I would feel’—was presented as a reasonable emotional response based on a gendered vulnerability, rather than a controversial racialized political stance. The scenario constructed respectable passivity, disrupted by the male asylum seekers being potential perpetrators of sexual violence in a safe public space—‘in my own town’ (Line 9).

This affective‐discursive privileged position (McConville et al., 2019) affords a claim of undeniable commonsense, which is underpinned by a fear of cultural differences and white supremacy (Bennett, 2025); Black and Brown men are likely to be potential perpetrators of sexual violence (Goodfellow, 2020). The speakers maintain a compassionate position that would welcome males, specifically categorized as dads arriving with their families and ‘kids’, because they are less prone to deviance in an isolated rural community. The local community is positioned as an entitled category that can determine who can reside in the bounded safe space, and whose fear is commonsense. The speakers were careful to inoculate against accusations of prejudice and maintained their compassionate position whilst implicitly constructing male asylum seekers as sexual predators. Avoiding explicit reference to race or culture, but working up a fear of idle unmarried men, without children, in an unlit, isolated rural area that lacks phone coverage, does the political work of presenting the protest as reasonable compassionate and warranted.

## CONCLUDING REMARKS

These protesters from this predominantly White, Irish and Catholic rural community used intimidation and arguably violent collective action to force asylum seekers out of their home and prevented additional asylum seekers from being housed. This may have led to them sleeping on the streets and being exposed to further violence (Johns, 2023; Power, 2023b; Wilson et al., 2023). However, it is notable that the protesters sanitized their justification for this action through a discursive strategy of compassionate exclusion.

They claimed they were concerned about the asylum seekers being housed in what they argue is unsuitable accommodation, primarily due to its rural location. They inoculated their actions against accusations of prejudice (Rapley, 2001), not through pragmatic structural and economic reasons, as shown in previous work (see Augoustinos & Every, 2007; Capdevila & Callaghan, 2008; Condor et al., 2006; Every & Augoustinos, 2007; Gibson et al., 2018; Goodman & Burke, 2010; Rapley, 2011; van Dijk, 1992, 1993b), but by claiming they are motivated by a concern for the welfare of the asylum seekers. This discursive strategy highlights how humanitarian concerns can be co‐opted to justify hostility towards asylum seekers (also see Every, 2008), and the ‘norm against prejudice’ affords discourses that legitimize forced exclusion (Billig, 1991).

The second commonly drawn‐upon discursive strategy was the lack of prior government consultation with the community. This also maintained the protesters' compassionate position and inoculated the justification for their actions against accusations of prejudice by directing the concern away from their explicitly hostile and arguably violent collective action to a rational liberal democratic concern of government failure. Notably, the discourse implicitly worked up a contradictory compassionate position, claiming that the protest would not happen and the community would not prevent asylum seekers from being housed if they had received prior consultation from the government. Nevertheless, the protesters were eventually consulted by the government, but they rejected an offer of mediation with the asylum seekers and a 4‐week freeze on additional residents, and the protest continued until the last of the asylum seekers were removed on 12 July 2024 (Cooke, 2024). In other words, government consultation did little to change the protesters' position. This discourse did less in the way of impression management and more in the way of rationalizing the protesters' actions by directing the attention away from their intimidation of the asylum seekers.

However, embedded within this benign request for prior consultation was the demand that potential residents should be vetted and the local community should be offered the decision on their removal and exclusion. They claimed they had the moral right to vet and exclude unwanted others, whom they constructed as being a threat to the women in the community (Goodfellow, 2020). Although previous work has shown how speakers employ fear of criminality (e.g., Figgou et al., 2011; Figgou & Condor, 2006) and inevitable intergroup conflict (Zisakou & Figgou, 2019) as a justification for excluding asylum seekers, these protesters specifically drew on gender as an issue for concern. While the protesters did not explicitly present the asylum seekers as potential sexual predators, the gender of the asylum seekers and women's vulnerability in a rural area without streetlights, footpaths and patchy phone coverage was made salient.

The specific threat to the community was never explicitly defined or evidenced, but unvetted men hanging around with little to do was presented as a commonsense threat to the women in this isolated rural community. The justification for the forced removal of these men was deracialized (Augoustinos & Every, 2007) by drawing on the specific category of the problematic ‘male’ asylum seekers (*cf*. Goodman & Speer, 2007; Lynn & Lea, 2003). And the protesters maintained a compassionate position by claiming they would welcome married men with children.

As highlighted in previous discursive work, rhetorically malleable social categories are deployed in novel ways to rationalize justifications for exclusion (Figgou et al., 2011; Figgou & Condor, 2007). Here, speakers drew on the legitimate concern about women's experience of sexual violence (Muldoon et al., 2023), but this was problematic because it orients towards a widespread toxic myth that immigrants are culturally barbaric (Goodfellow, 2020) and prone to committing sexual violence against White women (Chouhy & Madero‐Hernandez, 2020; Ogungbure, 2018). This discursive strategy worked up fear as an affective position to justify moral panic and a protest intended to intimidate and make the asylum seekers feel unwelcome.

However, the discourses drawn upon by the protesters were laced throughout with affect, including compassion and empathy for those whom they were demanding should be removed because they were fearful of them. This claim that ‘male’ asylum seekers were a threat to the women in the community was further inoculated against accusations of prejudice by working up a contradictory compassionate position towards the ‘lovely men’ who were already living in the accommodation. Evidently, speakers managed to take up an ambivalent affective position as both fearful and compassionate towards the asylum seekers. Similarly, Figgou et al. (2011) highlight that speakers can take up contradictory positions, disavowing the notion that immigrants are more likely to be involved in criminal activity as racist, whilst also presenting this taken‐for‐granted, unsubstantiated fear, that they did not necessarily agree with, as a justification for increased surveillance and exclusion of refugees.

These protesters presented their demand for the vetting of male asylum seekers as common sense, even though they provided no supporting evidence and counterintuitively presented the asylum seekers who were already housed at Magowna House as above suspicion and ‘lovely men’. This extends previous literature because this contradictory stance avoids accusations of prejudice but utilizes the malleable ‘norm against prejudice’ (Billig, 1991) to work up an arguably racist discourse as ‘not racist’ (Lentin, 2018). Lentin (2018) highlights that the rising hostility, systemic violence and popular racism towards asylum seekers is legitimized by restricting the definition of racism, which affords the hegemonic denial that this rising trend is not racist but based on legitimate concerns (also see Bennett, 2025; Goodfellow, 2020; Goodman, 2010; Waldinger, 2018). Whilst, as an extension of Billig's (1995) thesis of banal nationalism, which highlights that it is universally accepted that nations have the moral right to exclude unwanted others, these protesters positioned themselves as having category entitlement to determine who can reside in their community and who can be excluded based on a racialized myth and fear of the other (Bennett, 2025; Chouhy & Madero‐Hernandez, 2020; Figgou et al., 2011; Figgou & Condor, 2006).

Although these discourses undoubtedly work as a means of impression management for the media and listening audience, they also do considerable work justifying the collective action of the protesters as compassionate, rational and warranted. We do not interpret the discourse as a conduit to make inferences about internal mental states, but note that these speakers, who are engaged in this collective action to forcibly exclude unwanted others, paradoxically position themselves as compassionate towards those whom they want to exclude. Notably, explicit hostile discourses tended to be repressed (Billig, 1999) and were covertly couched in compassion, potentially because the discursive norm against prejudice shapes and facilitates this form of justification.

In sum, as far as we are aware, these discursive strategies of compassionate exclusion have not been previously documented. The protesters positioned themselves as compassionate towards the ‘lovely men’ whilst engaging in violent collective action to force the asylum seekers out of what they argue is unsuitable accommodation into homelessness and risk of further violence. They claimed they bore no hostility towards the ‘male’ asylum seekers, whilst demanding they should be vetted and the community should be consulted on their suitability for accommodation. Although we cannot generalize beyond this specific context, similar contradictory discourses may be evident in comparable Western liberal democracies where hostility and violence towards asylum seekers is increasing. It is evident that, although some discursive caution is required, the ‘norm against prejudice’ (Billig, 1991) affords readily available contradictory discursive strategies that can be effortlessly drawn upon to avoid the accusation of racism when justifying the forced removal and exclusion of unwanted others. Even though the discourses drawn upon sanitize the protesters' image, they also construct their action as rational and warranted by avoiding accusations of racism through a strategy of compassion for those whom they are violently hostile towards. Arguably, this compassionate justification for violent intimidation was facilitated by the narrow definition of racism to biological criteria and the norm against prejudice. This affords the ambivalent construction of a problematic category of ‘male’ asylum seekers who are also ‘lovely men’ and the concern for their welfare and lack of consultation is the premise for their forced removal and exclusion.

## AUTHOR CONTRIBUTIONS

**Alastair Nightingale:** Conceptualization; investigation; writing – original draft; methodology; validation; writing – review and editing; formal analysis; data curation; project administration. **Sarah Jay:** Investigation; writing – original draft; methodology; writing – review and editing; validation; formal analysis; data curation.

## CONFLICT OF INTEREST STATEMENT

The authors declare no conflict of interest.

## Supporting information


Table S1

## Data Availability

All the data for this article is publicly available online, and links have been provided in Table S1 in the Supplementary Material.
