# Supplementary material for: ‘They are lovely men’: Compassionate exclusion used to justify a protest outside asylum seeker accommodation
Source: Br J Soc Psychol. 2026 Feb 6;65(2):e70045. doi: 10.1111/bjso.70045 (PMC12879271; doi:10.1111/bjso.70045)
Supplement: Supplementary file 1 — Table S1 [file BJSO-65-0-s001.docx]

Supplementary Material: Data Table

| Date | Media platform | Duration | URL |
| --- | --- | --- | --- |
| 11/05/2023 | Clare FM News | 5:21 | <https://www.clare.fm/news/anger-lack-community-engagement-ahead-decision-accommodate-international-protection-applicants-inch-hotel-3/> |
| 15/05/2023 | Clare FM | 3:31 | <https://soundcloud.com/clarefm/west-clare-councillor-claims-asylum-seekers-will-be-virtually-imprisoned-at-magowna-house> |
| 16/05/2023 | RTE Radio One Morning Ireland | 6:25 | <https://www.rte.ie/radio/radio1/clips/22250703/> |
| 16/05/2023 | RTE Radio One Morning Ireland | 13:02 | <https://www.rte.ie/radio/radio1/clips/22250893/> |
| 16/05/2023 | RTE Radio Drivetime | 13:11 | <https://www.rte.ie/radio/radio1/clips/22251354/> |
| 17/05/2023 | Clare FM News | 10:00 | <https://soundcloud.com/clarefm/minister-confirms-magowna-house-emergency-accommodation-will-remain-in-place> |
| 17/05/2023 | Clare FM News | 9:00 | <https://www.clare.fm/news/clare-immigrant-support-centre-defends-use-inch-hotel-accommodating-international-protection-applicants-3/> |
| 17/05/2023 | RTE Radio Morning Ireland | 7:45 | <https://www.rte.ie/radio/radio1/clips/22251411/> |
| 17/05/2023 | RTE Radio Morning Ireland | 12:15 | <https://www.rte.ie/radio/podcasts/22251507-co-clare-politicians-to-meet-min-roderic-ogorma/> |
| 17/05/2032 | RTE News | 2:03 | <https://www.rte.ie/news/munster/2023/0517/1384019-clare-hotel/> |
| 17/05/2023 | RTE News | 1:05 | <https://www.rte.ie/news/ireland/2023/0517/1384079-asylum-seekers-reax/> |
| 17/05/2023 | RTE Radio News at One | 4:37 | <https://www.rte.ie/radio/radio1/clips/22251832/> |
| 17/05/2023 | RTE Radio Drivetime | 17:34 | <https://www.rte.ie/radio/podcasts/22251943-magowna-house-blockade-latest/> |
| 17/05/2023 | RTE Radio The Late Debate | 18:10 | <https://www.rte.ie/radio/podcasts/22251925-political-reaction-to-the-blockade-in-clare-today/> |
| 18/05/2023 | RTE Morning Ireland | 10:25 | <https://www.rte.ie/radio/radio1/clips/22252015/> |
| 18/05/2023 | RTE Radio Drivetime | 13:35 | <https://www.rte.ie/radio/podcasts/22252497-talks-ongoing-to-end-the-clare-blockade/> |
| 18/05/2023 | Clare FM | 16:43 | <https://www.clare.fm/news/finding-common-ground-key-bringing-magowna-protest-end-3/> |
| 18/05/2023 | Clare FM News | 2:28 | <https://www.clare.fm/news/inch-residents-say-peaceful-protest-will-continue-government-overturns-magowna-decision-3/> |
| 19/05/2023 | RTE Radio Morning Ireland | 3:26 | <https://www.rte.ie/radio/radio1/clips/22252790/> |
| 19/05/2023 | RTE Radio Drivetime | 10:23 | <https://www.rte.ie/radio/podcasts/22253121-inch-locals-say-they-will-maintain-their-blockade/> |
| 19/05/2023 | RTE News | 6:24 | <https://www.rte.ie/news/munster/2023/0518/1384329-clare-asylum-hotel/> |
| 19/05/2023 | The Irish Times In The News | 24:50 | <https://www.irishtimes.com/ireland/2023/05/20/inch-migrant-blockade-i-heard-a-woman-shouting-we-dont-want-them-here-it-was-not-subtle/> |
| 19/05/2023 | Clare FM | 9:05 | <https://soundcloud.com/clarefm/inch-protestors-feel-government-proposal-is-disingenious> |
| 21/05/2023 | RTE Radio This Week | 4:36 | <https://www.rte.ie/radio/podcasts/22253259-protestors-in-clare-lift-blockade-on-hotel-used-fo/> |
| 21/05/2023 | The Irish Times | 4:19 | <https://www.irishtimes.com/ireland/social-affairs/2023/05/21/blockade-lifted-at-asylum-seeker-centre-in-co-clare-as-ogorman-says-protest-not-appropriate/> |
| 22/05/2023 | Clare FM | 4:25 | <https://soundcloud.com/clarefm/clare-senator-hopeful-removal-of-magowna-blockade-is-beginning-of-positive-end> |
| 23/05/2023 | Clare FM News | 18:57 | <https://www.clare.fm/news/minister-says-state-engaging-inch-community-diffuse-tensions-3/> |
| 24/05/2023 | Clare FM | 11:33 | <https://soundcloud.com/clarefm/taoiseach-acknowledges-communication-with-clare-communities-should-have-been-better> |
| 25/05/2023 | Clare FM | 10:55 | <https://soundcloud.com/clarefm/empathy-advocate-confident-succesful-integration-can-be-achieved-in-any-community> |
| 26/05/2023 | Clare FM | 1:11 | <https://soundcloud.com/clarefm/assistant-commissioner-denies-reports-gardai-allowed-protestors-onto-bus-at-magowna-house> |
| 14/06/2023 | Clare FM | 2:29 | <https://soundcloud.com/clarefm/north-clare-councillor-claims-succesful-inegration-only-achievable-with-clear-dialogue> |
| 16/06/2023 | Clare FM | 6:06 | <https://www.clare.fm/news/clare-independent-td-claims-respect-inch-communitys-needs-vital-solve-magowna-impasse-3/> |
| 16/06/2023 | Clare FM | 10:45 | <https://www.clare.fm/news/clare-independent-td-claims-respect-inch-communitys-needs-vital-solve-magowna-impasse-3/> |
| 20/06/2023 | Clare FM | 2:05 | <https://www.clare.fm/news/inch-community-group-agree-mediation-process-aimed-resolving-magowna-house-impasse-3/> |
|  |  |  |  |
|  | Total | 4:58:39 |  |
